# How to Count Our Microbes? The Effect of Different Quantitative Microbiome Profiling Approaches

**DOI:** 10.3389/fcimb.2020.00403

**Published:** 2020-08-07

**Authors:** Gianluca Galazzo, Niels van Best, Birke J. Benedikter, Kevin Janssen, Liene Bervoets, Christel Driessen, Melissa Oomen, Mayk Lucchesi, Pascalle H. van Eijck, Heike E. F. Becker, Mathias W. Hornef, Paul H. Savelkoul, Frank R. M. Stassen, Petra F. Wolffs, John Penders

**Affiliations:** ^1^Department of Medical Microbiology, School of Nutrition and Translational Research in Metabolism (NUTRIM), Maastricht University Medical Center+, Maastricht, Netherlands; ^2^Department of Medical Microbiology, Care and Public Health Research Institute (CAPHRI), Maastricht University Medical Center+, Maastricht, Netherlands; ^3^Institute of Medical Microbiology, RWTH University Hospital Aachen, Aachen, Germany; ^4^Member of the German Center for Lung Research (DZL), Universities of Giessen and Marburg Lung Centre, Institute for Lung Research, Philipps-University Marburg, Marburg, Germany; ^5^Department of Respiratory Medicine, School of Nutrition and Translational Research in Metabolism (NUTRIM), Maastricht University Medical Center+, Maastricht, Netherlands; ^6^Division of Gastroenterology-Hepatology, Department of Internal Medicine, School of Nutrition and Translational Research in Metabolism (NUTRIM), Maastricht University Medical Center+, Maastricht, Netherlands; ^7^Department of Medical Microbiology and Infection Control, VU University Medical Center, Amsterdam, Netherlands

**Keywords:** digital droplet PCR, microbiota, viability PCR, 16S rRNA gene, flow cytometry, quantitative PCR

## Abstract

Next-generation sequencing (NGS) has instigated the research on the role of the microbiome in health and disease. The compositional nature of such microbiome datasets makes it however challenging to identify those microbial taxa that are truly associated with an intervention or health outcome. Quantitative microbiome profiling overcomes the compositional structure of microbiome sequencing data by integrating absolute quantification of microbial abundances into the NGS data. Both cell-based methods (e.g., flow cytometry) and molecular methods (qPCR) have been used to determine the absolute microbial abundances, but to what extent different quantification methods generate similar quantitative microbiome profiles has so far not been explored. Here we compared relative microbiome profiling (without incorporation of microbial quantification) to three variations of quantitative microbiome profiling: (1) microbial cell counting using flow cytometry (QMP), (2) counting of microbial cells using flow cytometry combined with Propidium Monoazide pre-treatment of fecal samples before metagenomics DNA isolation in order to only profile the microbial composition of intact cells (QMP-PMA), and (3) molecular based quantification of the microbial load using qPCR targeting the 16S rRNA gene. Although qPCR and flow cytometry both resulted in accurate and strongly correlated results when quantifying the bacterial abundance of a mock community of bacterial cells, the two methods resulted in highly divergent quantitative microbial profiles when analyzing the microbial composition of fecal samples from 16 healthy volunteers. These differences could not be attributed to the presence of free extracellular prokaryotic DNA in the fecal samples as sample pre-treatment with Propidium Monoazide did not improve the concordance between qPCR-based and flow cytometry-based QMP. Also lack of precision of qPCR was ruled out as a major cause of the disconcordant findings, since quantification of the fecal microbial load by the highly sensitive digital droplet PCR correlated strongly with qPCR. In conclusion, quantitative microbiome profiling is an elegant approach to bypass the compositional nature of microbiome NGS data, however it is important to realize that technical sources of variability may introduce substantial additional bias depending on the quantification method being used.

## Introduction

Next-generation sequencing (NGS) has instigated microbiome research and resulted in many novel insights on the role of the microbiome in health and disease. One of the challenges of NGS however relates to the compositional nature of the generated data. As compositional data always sum up to a constant (e.g., 100%), an increase of a specific microbial taxon in response to a given condition will inevitably lead to a decrease in the relative abundance of other taxa. This mutual dependence between microbial taxa when expressed as relative abundances makes it particularly challenging to identify those microbial taxa that are truly affected by an intervention or a disease state (Knight et al., 2018; Jian et al., 2020).

Vandeputte et al. (2017) introduced the concept of Quantitative Microbiome Profiling (QMP) as a way to quantify absolute microbial abundances from NGS data to bypass many of the statistical and interpretative challenges that arise from the compositional structure of microbiome sequencing data. In their work, QMP was achieved by determining the total bacterial load of stool samples by flow-cytometry and subsequently normalizing the 16S rRNA gene sequencing data for sampling depth taking the total bacterial cell counts into account. In contrast, Jian et al. (2020) used quantitative PCR (qPCR) as a simple and cost-effective alternative to determine the bacterial load and estimate the absolute taxon abundance from NGS data.

Both cell-counting and qPCR come with their advantages and limitations which can impact the subsequent estimation of absolute taxon abundances. Flow-cytometry counts only intact microbial cells. Therefore, new bias could theoretically be introduced when samples contain a significant amount of free extracellular prokaryotic DNA. This free DNA is captured during sequencing but is excluded during flow-cytometry cell counting. In case the taxonomic composition of free circulating DNA differs from the composition of intact microbial cells (e.g., due to differences in the resistance of microbial cells to environmental stress), this might result in the introduction of a new source of bias in downstream analysis. Enumerating bacteria on the basis of qPCR would introduce biases through the extraction, purification, and amplification of DNA. Although, one could argue that this also applies to the NGS data and as such could be considered an advantage of qPCR-based quantification (Jian et al., 2020). Advantages of qPCR-based quantification are the cost-effectiveness, simplicity and accessibility, whereas the sensitivity might be a limitation as qPCR has been reported to be only sensitive enough to detect 2-fold changes in gene concentration or microbial load (Smith and Osborn, 2009).

Although Vandeputte et al. (2017) showed only a moderate correlation between quantification of microbial load by flow-cytometry and qPCR, a direct comparison between cell-based and molecular-based methods to estimate absolute taxon abundances from NGS data has not yet been conducted. As such the level of potential bias that could additionally be introduced when applying quantitative microbial profiling remains unknown.

Here we explored both cell-based and molecular-based methods for QMP and examined the potential effect of various sources of bias by analyzing the fecal microbial profiles of 16 healthy volunteers.

First, we compared the estimation of absolute microbial taxon abundances by combining 16S rRNA gene amplicon profiling with, respectively, flow-cytometry and qPCR to determine the microbial load.

Second, we examined to what extend extracellular DNA derived from lysed bacteria might introduce differences between cell-based and molecular-based QMP approaches by eliminating free DNA and non-viable cells from stool samples using Propidium Monoazide (PMAxx™, Biotium, Fremont, CA, USA) treatment. Last, we compared the (lack of) sensitivity of qPCR-based methods for microbial quantification to digital droplet PCR (Hindson et al., 2013) as a more precise, discriminating, and reproducible molecular quantification method (Kim et al., 2014; Gobert et al., 2018).

## Materials and Methods

### Study Population

To assess the impact of different quantitative microbial profiling methods, we collected fecal samples from 16 healthy volunteers. Along with sample collection, a limited number of demographic data were retrieved, including date and time of fecal collection, age, sex, dietary lifestyle, and antibiotic consumption in the previous 3 months (Table 1).

**Table 1:** Characteristics of the (fecal samples of) healthy subjects included in the present study.

**Subject**	**Age**	**Sex**	**Alternative dietary lifestyle**	**Antibiotic use in past 3 months**	**Bristol stool score**	**Average % dry weight**
1	25	Female	Vegetarian	No	3	26.99
2	24	Male	No	No	3	18.70
3	28	Female	Vegetarian	No	6	23.43
4	23	Female	No	No	4	28.08
5	31	Female	No	No	4	23.34
8	29	Female	No	No	6	18.52
9	27	Female	No	No	3	36.29
10	49	Female	No	No	3	14.73
12	30	Male	No	No	4	26.82
13	27	Male	No	No	4	22.47
15	29	Female	No	No	4	13.00
16	26	Female	No	No	6	16.37
20	26	Male	No	No	3	24.49
22	26	Male	No	No	3	24.58
23	31	Male	No	No	4	29.89
26	31	Male	No	No	4	19.56

Participants were instructed to collect a complete defecation in a FecesCatcher (Tag Hemi VOF, Zeijen, The Netherlands), transfer a maximum amount of feces in a labeled feces tube (Sarstedt, Nümbrecht, Germany) and deposit the sample and accompanying questionnaire in a sealed plastic safety bag at the research department as soon as possible. All samples were aliquoted (200 mg aliquots) and stored at −80°C by the researchers.

### Cell Counts and Stool Moisture

For cell counting, 200 mg aliquots of the samples were processed and stained as described by Vandeputte et al. (2017) followed by flow cytometric analysis using a BD FACSCanto II with FACS Diva V8.0.1 software (BD Biosciences). A side scatter of 2,000 was set as acquisition threshold. All other instrument and gating settings were in accordance with the method described by Vandeputte et al. (2017) and were kept constant for all samples. To obtain bacterial concentrations, the total number of events in the cell gate was divided by the sample volume, which was determined by weighing each tube before and after acquisition.

Stool moisture content was determined in duplicate on 200 mg homogenized fecal material as the percentage of mass loss upon vacuum concentration for 5 h at 60°C in a Vacufuge plus (Eppendorf) using the “AQ” setting.

### PMAxx Treatment

For the QMP-PMA approach, extracellular DNA and DNA from dead or membrane-compromised bacterial cells was removed by pre-treatment of fecal samples with the viability dye PMAxx™. PMAxx is a DNA-intercalating agent that forms photo-induced crosslinks making the bound DNA inaccessible for downstream molecular applications. PMAxx was added to 10-fold diluted fecal specimens at a final concentration of 50 μM, followed by 10 min. shaded incubation at 4°C. Photoactivation was performed by using the PMA-Lite™ LED Photolysis Device (Biotium) with the exposure time set to 10 min. This procedure was repeated 3 times after which metagenomic DNA was isolated from the samples.

In order to assess the effectiveness of PMA-treatment, three fecal samples were spiked with 3.7 × 10^7^ copies/gram feces of heat-killed *Chlamydia trachomatis* (CT). Subsequently, samples were split in two aliquots of which one aliquot was treated with PMAxx as described above and one aliquot remained untreated. Upon DNA isolation (see below), the CT load was quantified by subjecting the treated and untreated samples to a qPCR assay targeting the single-copy *ompA* gene, coding for the major outer membrane protein (MOMP) of *C. trachomatis*, on a 7900HT Real-Time PCR System (Applied Biosystems, Foster City, California) as described previously (Janssen et al., 2016).

### DNA Isolation and qPCR Assessment of Bacterial Load

DNA was extracted from 200 mg of frozen aliquots of homogenized feces according to the recommended protocol Q of the International Human Microbiome Standards Consortium (Costea et al., 2017).

Extracted DNA was quantified using a Qubit 2.0 Fluorometer (Thermo Fisher Scientific).

Enumeration of total bacterial load by qPCR was achieved by amplification of the 16S rRNA genes (primer pair 16S-341_F and 16S-805_R; CCTACGGGNGGCWGCAG and GACTACHVGGGTATCTAATCC, respectively) using a MyiQ Single-Color Real-Time PCR Detection System (BioRad) in 25 μl reactions containing 12.5 μl iQ SYBR Green Supermix (BioRad), 2 μl template DNA (1:1000 diluted), 300 nM of both primers 16S-341_F and 16S-805_R. The PCR amplification program consisted of an initial denaturation set at 95°C for 3 min. followed by 35 three-step cycles at 95°C for 15 s and at 55°C for 20 s and 72°C for 30 s. In each run, negative template controls (DNA replaced by nuclease-free water in qPCR), negative isolation controls (feces replaced by nuclease free water during DNA extraction) and positive controls (quantified recombinant plasmid construct containing the target sequence) were included. Melting curves were checked for each sample to confirm amplification of the correct product.

### Digital Droplet PCR for Assessment of Bacterial Load

Next to molecular quantification by qPCR, all samples were also quantified by ddPCR by amplifying the 16S rRNA gene (primer pair 515F/806R; Caporaso et al., 2012) using a QX200 Droplet Digital PCR system (Bio-rad). Reaction mixtures consisting of 11 μl EvaGreen ddPCR Supermix (Bio-Rad), 2.2 μl template DNA and 300 nM of both primers in 22 μl reaction volumes were prepared and 20 μl will be transferred to the DG8 droplet generator cartridge. Upon the addition of 70 μl Droplet Generation Oil in the dedicated wells, the cartridge was placed in the QX200 droplet generator. After droplets have been generated, 40 μl was transferred to a 96-wells PCR plate and the plate was sealed using a PX1 PCR plate sealer. The PCR amplification program consisted of an initial denaturation set at 95°C for 3 min. followed by 30 three-step cycles at 95°C for 30 s and at 50°C for 45 s and 72°C for 1 min and finally followed by post-cycling steps of 98°C for 10 min (enzyme inactivation) and an infinite 12°C hold. The plate was subsequently placed in a QX200 droplet reader and results were analyzed using the Quantasoft application.

### Comparison of Cell-Based and Molecular-Based Quantification of a Standard Microbial Community

We used the Gut Microbiome Whole cell Mix (ATCC® MSA-2006^TM^) containing an even mixture of whole bacterial cells (12 different species) in order to assess whether cell-based or molecular-based quantification was more accurate. The lyophilized pellet was dissolved in 1 ml PBS according to the manufacturer's instructions and serial 2-fold dilutions, ranging from 3.3 × 10^6^ to 5.56 × 10^4^, were subsequently made. The dilutions were used for cell counting as well as for DNA-isolation followed by qPCR as described above. For qPCR, the number of copies/ml were converted into cells/ml by taking into account the copy numbers for each of the bacterial species in the mock community (average copy number 6.435/genome).

### Microbiota Profiling

Fecal microbiota profiling was performed in accordance to the paper by Vandeputte et al. (2017).

Briefly, the V4 region of the 16S rRNA gene was PCR amplified from each DNA sample in triplicate using the 515F/806R primer pair described previously (Caporaso et al., 2012). Pooled amplicons from the triplicate reactions were purified using AMPure XP purification (Agencourt) according to the manufacturer's instructions and eluted in 25 μl 1 × low TE (10 mM Tris-HCl, 0.1 mM EDTA, pH 8.0). Quantification of amplicons was subsequently performed by the Quant-iT PicoGreen dsDNA reagent kit (Invitrogen) using a Victor3 Multilabel Counter (Perkin Elmer, Waltham, USA). Amplicons were mixed in equimolar concentrations to ensure equal representation of each sample and sequenced on an Illumina MiSeq instrument (MiSeq Reagent Kit v3, 2 × 250 cycles, 10% PhiX) to generate paired-end reads of 250 bases in length in both directions.

After demultiplexing using MiSeq reporter software using default settings, fastq sequences were merged, quality and chimera filtered using FLASH (Magoc and Salzberg, 2011), seqtk trimq (https://github.com/lh3/seqtk) and usearch (Edgar et al., 2011), respectively, using the same settings as Vandeputte et al. (2017).

Finally, between 153,527 and 282,297 reads per untreated sample and between 152,968 and 268,362 reads per PMAxx-treated samples remained for downstream analysis.

#### Relative Microbiome Profiling (RMP)

Samples were downsized to 153,527 reads/sample by randomly selecting reads. Taxonomic assignment of reads was performed using RDP classifier 2.12 (Wang et al., 2007).

#### Cell-Based Quantitative Microbiome Profiling (QMP)

QMP was done in accordance with the method proposed by Vandeputte et al., downsizing the samples to an even sampling depth, defined as the ratio between sample size (16S rRNA gene copy-number-corrected sequencing depth) and microbial load (average total cell count/gram frozen feces; Table S3).

#### PMA-Based Quantitative Microbiome Profiling (QMP-PMA)

Quantitative microbiome profiling after removal of extracellular DNA and DNA from dead and damaged bacterial cells was conducted identical to the standard QMP method with the exception of the additional PMA pre-treatment prior to metagenomic DNA-isolation.

#### qPCR-Based Quantitative Microbiome Profiling (QMP-qPCR)

The bacterial load was determined by qPCR targeting the 16S rRNA gene. Comparing cycle threshold values of each sample to a standard quantification curve (using quantified recombinant plasmid constructs) resulted in the total number of 16S rRNA gene copies/gram feces (Table S4). In order to use the qPCR-based determination of bacterial load, total numbers of 16S rRNA gene copies/gram feces were converted into the total number of bacterial cells/gram feces. First, the average number of 16S rRNA gene copies per bacterium was calculated for each sample based upon the sequencing data (total number of sequencing reads for a given sample divided by the copy-number corrected number of reads for that respective sample). Next, the total number of 16S rRNA gene copies/gram feces as determined by qPCR was divided by the average 16S rRNA gene copy number of that respective sample. Subsequently, the same approach as for the standard QMP method was followed.

### Statistical Analyses

No sample size calculations were performed. Statistical analyses were performed in R using the packages vegan (Oksanen et al., 2013) and DirichletMultinomial (Morgan, 2017). Two sided statistical tests were used for all comparisons and corrected for multiple testing using the false discovery rate [FDR according to Benjamini-Hochberg method (Benjamini and Hochberg, 1995)] where appropriate.

*Observed genus richness* was calculated using the R package vegan and *enterotyping* using the DMM approach was performed in R as described previously (Holmes et al., 2012). As the DMM-clustering was based on a limited number of samples, hence having potentially a limited accuracy, we examined whether the clustering was in concordance with the classification according to the reference-based enterotype classification model fitted on MetaHIT samples (enterotypes.org) (Costea et al., 2018) DMM-based clustering and reference-based classification was in accordance for all samples, with the exception of three samples that were classified into the Firmicutes enterotype according to the classification model. To calculate microbiome variation between replicates and methods, the *Bray–Curtis dissimilarity* based on the genus-abundance matrix was calculated and visualized by PCoA using the vegan package.

Pearson's or, where appropriate, non-parametric Spearman's correlations were calculated to determine the association between continuous variables (genus richness and abundances, bacterial load, and/or metadata).

Paired Wilcoxon Signed Rank test was used to test for differences in observed genus richness between profiling methods and to test for differences in microbiome variation between replicates and profiling methods. The Mann–Whitney U-test was used to test for differences in bacterial loads between enterotypes.

To calculate the ordinal association between genera in the four different profiling methods, the Kendall rank correlation coefficient was used to test for concordance of ranking for the 15 most abundant genera (based upon RMP) between the methods.

## Results and Discussion

To examine the correlation between cell-counting and molecular quantification, we first compared the quantification of the serially diluted Gut Microbiome Whole cell Mix by means of flow cytometric and qPCR. Cell counting resulted in a concentration very similar to the expected concentration as provided by the manufacturer as quantified by a cellometer (Table S1). Although quantification of serial 2-fold dilutions of the cell mix by FACS and qPCR correlated very strongly (Pearson's *r* = −0.967, *P* = 1.7 × 10^−8^, Table S1), qPCR resulted in a much higher than expected concentration (1.0 × 10^8^ cells/ml). Since qPCR also detects extracellular DNA, while cell-counting only quantifies intact microbial cells, we next removed extracellular DNA by pre-treatment of the suspended Gut Microbiome cell Mix with the viability dye PMAxx™ prior to metagenomic DNA isolation. After PMAxx™ pre-treatment, the number of bacterial cells in the mix as quantified by qPCR was 3.57 × 10^6^ cells/ml, and thereby almost identical to the expected concentration. Also, after PMAxx™ pre-treatment, the correlation between cell counting and qPCR of serially diluted Gut Microbiome cell Mix remained very strong (Pearson's *r* = −0.966, *P* = 2.1 × 10^−8^, Table S1). Altogether these results indicate that flow cytometry-based cell counting, and qPCR-based quantification correlated strongly, but absolute quantification might differ substantially in the presence of large quantities of extracellular DNA.

**Figure 1:**
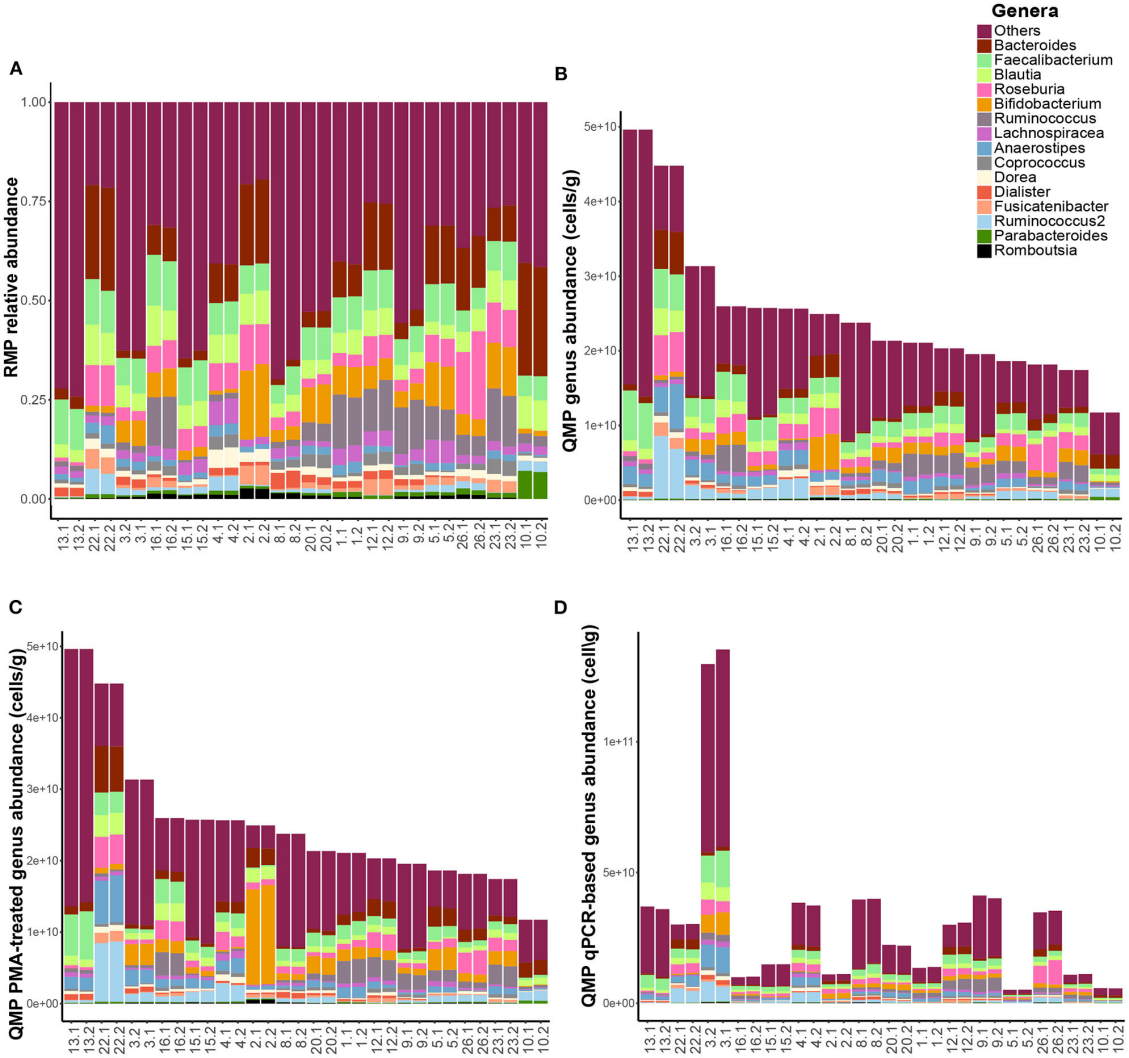
Microbiome profile comparisons. Genus-level fecal microbial composition of both replicates of all 16 healthy study subjects (*n* = 32 samples) based upon **(A)** relative microbiome profiling (RMP), **(B)** quantitative microbiome profiling (QMP, cells per gram feces), **(C)** QMP after PMAxx-treatment of fecal samples (QMP-PMA, cells per gram feces), and **(D)** QMP using qPCR for quantification of bacterial load (QMP-qPCR, cells per gram feces).

Next, we profiled the microbiota of fecal samples of the 16 healthy volunteers in duplicate for each of the four methods: (i) Relative Microbial Profiling (RMP), (ii) Quantitative Microbial Profiling using flow cytometry-based microbial load (QMP), (iii) Quantitative Microbial profiling using flow-cytometry-based microbial load and PMAxx™ pre-treatment before metagenomics DNA isolation and 16S rRNA gene amplicon sequencing in order to only profile the microbial composition of intact cells (QMP-PMA), and (iv) Quantitative Microbial Profiling using qPCR to determine the microbial load (QMP-qPCR) (see **Methods** section for details, Table 1 for study-specific data and Figures 1A–D for microbial profiles).

Stool moisture negatively correlated with observed richness (Spearman's ρ = −0.685, FDR = 9.0 × 10^−3^, Table S2) confirming previous observations between stool consistency and microbial richness (Tigchelaar et al., 2016; Vandeputte et al., 2016, 2017) A similar correlation with microbial richness was not observed when using the Bristol Stool Scale (BSS) as a measure for stool consistency (Spearman's ρ = −0.15, FDR = 5.9 × 10^−1^). Indeed, BSS scores only weakly and non-significantly correlated with stool moisture (Spearman's ρ = 0.27, FDR = 4.6 × 10^−1^). This lack of correlation is likely the result of the potential bias introduced by the self-reporting of BSS scores by the study participants, advocating the standardized scoring of stool consistency by research staff or using more objective markers such as stool moisture (Vork et al., 2019).

Microbial loads as assessed by flow-cytometry were shown to vary between 1.2 × 10^10^ and 5.3 × 10^10^ cell counts per gram of fecal material (median 2.3 × 10^10^ cell counts per gram; Table S3) and a comparison with qPCR enumeration revealed a moderate correlation (Pearson's *r* = −0.50, *P* = 4.7 × 10^−2^, Table S3, Figure S1) similar to what has been described by Vandeputte et al. (2017).

Using DMM clustering on RMP profiles, we identified two enterotypes enriched in *Bacteroides* or *Prevotella* (Figure S2). The microbial loads, as determined by flow-cytometry, significantly differed between the two enterotypes (median 1.91 × 10^10^ and 2.43 × 10^10^ cells/gram, respectively, *P* = 4.4 × 10^−2^). A similar difference between enterotypes was, however, absent when the microbial loads were determined by qPCR (*P* = 6.0 × 10^−1^, Table S4).

Prior to comparing the QMP- and QMP-PMA data, we examined the efficacy of PMAxx treatment in removing extracellular DNA in a fecal matrix. First, spiking of fecal samples with heat-killed *C. trachomatis* (CT) showed that PMAxx treatment effectively eliminated free DNA as indicated by a substantial reduction of qPCR detection of the CT-target DNA (i.e., average increase of 11.6 Ct-values (range 10.2–12.7) in qPCR which is equivalent to a signal reduction of 99.96%). Second, enumeration of total bacterial load in fecal samples by qPCR revealed an average decrease in bacterial load of 1.5 × 10^10^ 16S rRNA gene copies/gram feces [IQR 5.1 × 10^9^-2.7 × 10^10^, *P* = 5.2 × 10^−4^, Figure S3] upon PMAxx treatment corresponding to an average of 39.0% of metagenomic DNA being extracellular or originating from non-viable cells. However, the correlation between microbial loads as assessed by flow-cytometry and qPCR appeared to be slightly weaker after PMAxx-treatment (Pearson's *r* = −0.41, *P* = 1.1 × 10^−1^, Table S3, Figure S1).

Generating quantitative microbiome profiles revealed that profiles obtained after PMAxx-treatment remained highly similar to the standard QMP profiles (Figures 1B,C), although the observed genus richness slightly decreased upon PMAxx-treatment (median richness 66.0 and 64.0 for QMP and QMP-PMA, respectively, FDR = 4.0 × 10^−3^, Table S2). Determination of bacterial load by qPCR, however, resulted in highly divergent profiles (Figure 1D) and a strong decrease in the observed genus richness (median: 52.0, FDR = 1.2 × 10^−3^) when compared to QMP and QMP-PMA.

**Figure 2:**
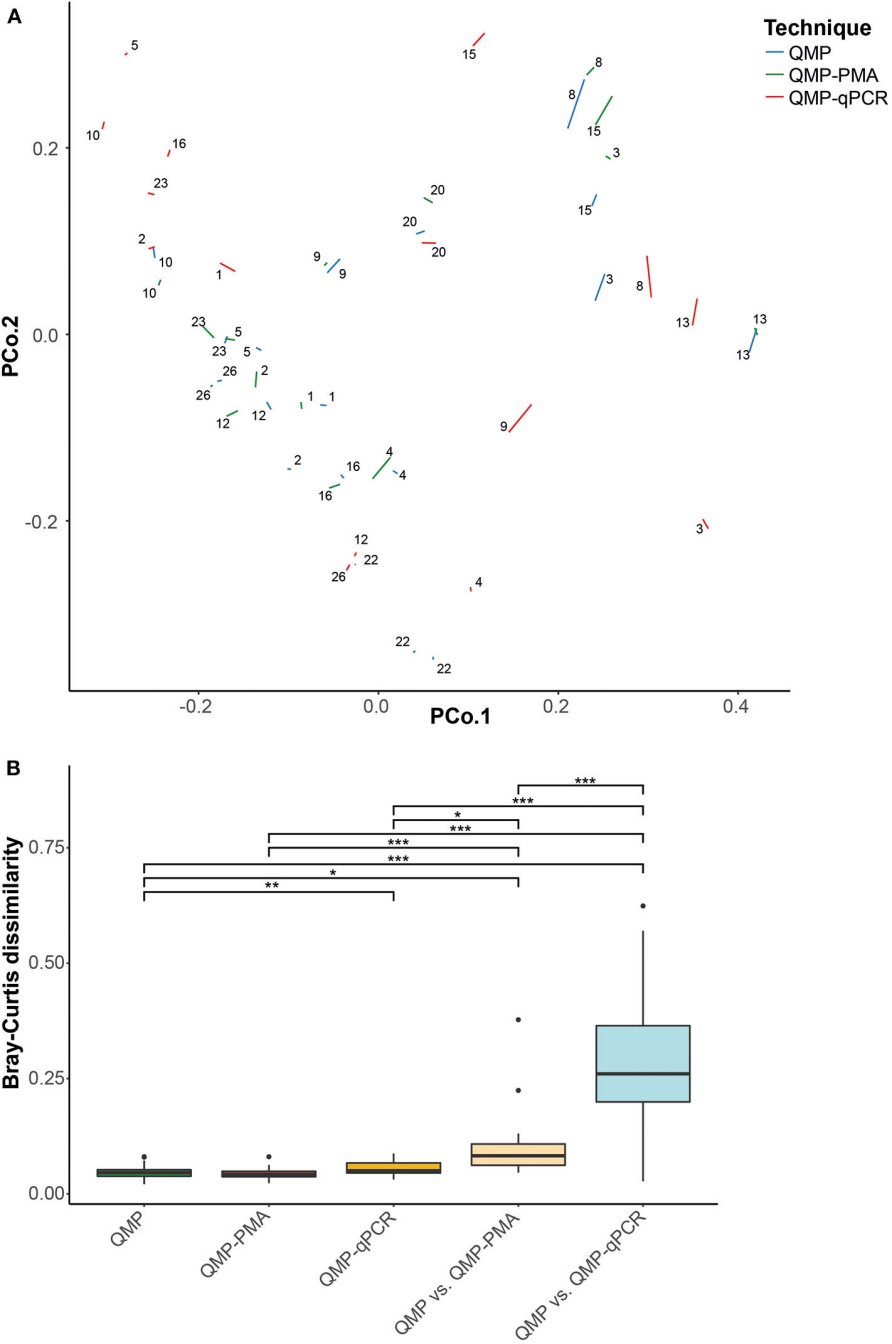
Within method dissimilarity of sample replicates and between methods dissimilarity of samples. Fecal microbial community structure variation based upon Bray–Curtis (BC) dissimilarity between samples and sample replicates. **(A)** Principal coordinates analysis of the study cohort based upon BC dissimilarity. Each segment connects the two replicates of the same sample as profiled by QMP (blue), QMP-PMA (green), and QMP-qPCR (red), **(B)** Box-plot of BC distance between sample replicates for all quantitative profiling methods (within-method variability) and BC distance in microbial community structure from the same sample profiled with different quantitative methods (between-method variability). The significance was checked pairwise using the Wilcoxon test and then adjusted for multiple comparisons using the FDR correction. The significance coding is indicated as ***p < 0.005, **p < 0.01, *p < 0.05 and N.S. for *p* ≥ 0.05. For clarity only significance of the comparisons between within QMP-method dissimilarity and all other within- and between-method dissimilarities are indicated (all FDR-corrected *p*-values are presented in Table S5).

We subsequently analyzed the divergence in microbial community structure both between replicates of samples analyzed by the same QMP method (within-method dissimilarity) as well as between aliquots of the same sample but profiled by different quantitative methods (between-methods dissimilarity). The within-method variation, as indicated by the average Bray Curtis (BC) dissimilarity, was similar for QMP with and without PMAxx-treatment (Figure 2, Table S5, FDR = 5.62 × 10^−1^), whereas the within-method variation was slightly higher for QMP-qPCR when compared to the standard QMP method (FDR = 9.66 × 10^−4^). Although the between QMP and QMP-PMA method dissimilarity was significantly larger than the within QMP-method dissimilarity (FDR = 1.44 × 10^−3^), the dissimilarity in microbial community structure between both methods was still modest [median (IQR) BC dissimilarity: 0.082 (0.062–0.108)] and far lower than the dissimilarity between QMP-qPCR and QMP [median (IQR): 0.260 (0.199–0.364), FDR = 1.83 × 10^−4^]. From these results it cannot yet be deduced whether the slightly yet significantly dissimilar QMP-PMA and QMP microbial profiles are due to the elimination of free extracellular DNA (bias in QMP) or merely due to the introduction of additional technical variation during sample handling (noise).

We therefore subsequently examined to what extent the sample rank order for each genus was conserved between the four profiling methods, similar to Vandeputte et al., When comparing RMP to QMP, sample rank order concordance within the 15 most abundant genera varied widely with the highest concordance observed for *Fusicatenibacter* and the lowest concordance for *Blautia* (Kendall's rank correlation test, τ, range = 0.47–0.95, Table S6). This confirmed the previous observation that absolute abundance profiles differ significantly from those generated by relative approaches.

When comparing the average sample rank concordance among the 15 most abundant genera between each of the four profiling methods, QMP and QMP-PMA showed the highest overall concordance (average τ among the 15 most abundant genera = 0.82, Table S6, Figure S4). The overall concordance between RMP and either QMP or QMP-PMA did not differ significantly (average τ among 15 most abundant genera = 0.75 and 0.69, respectively, FDR = 3.8 × 10^−1^, Table S6, Figure S4), indicating that PMAxx-treatment did not appear to result in a higher overall concordance with RMP. For each of the 15 genera the lowest sample rank order concordance was observed between QMP-qPCR and the other three methods, confirming that qPCR-based absolute abundance profiles are highly divergent from both the other quantitative as well as the relative profiling methods.

Furthermore, we could clearly identify the strong trade-off between *Bacteroides* and *Prevotella* as commonly reported (Lozupone et al., 2012) in RMP-based analysis (Spearman's ρ = −0.70, FDR = 3.2 × 10^−5^) and confirmed that the association between these two genera became weaker in a quantitative context, although the association remained statistically significant in the QMP and QMP-PMA profiles (QMP: Spearman's ρ = −0.64, FDR = 1.7 × 10^−4^; QMP-PMA: Spearman's ρ = −0.55, FDR = 1.4 × 10^−3^; QMP-qPCR: Spearman's ρ = −0.17, FDR = 0.353; Table S7).

To explore the possibility that the deviant profiles generated by QMP-qPCR are the result of the lack of precision and sensitivity of qPCR-based quantification, we finally quantified the microbial load in all fecal samples by means of Droplet Digital PCR (ddPCR). As with qPCR, this more recently introduced technology uses Taq polymerase in a standard PCR reaction to amplify the target DNA. The ddPCR technology however partitions the PCR reaction into thousands of droplets (individual reaction vessels) prior to amplification and acquires the data at the reaction end point. This enables more precise and reproducible data and direct quantification without the need of standard curves (Kim et al., 2014; Gobert et al., 2018). Quantification of microbial load based upon ddPCR however correlated strongly with qPCR-based quantification both for untreated (Pearson's *r* = 0.72, *P* = 2.0 × 10^−3^) and PMAxx-treated fecal samples (Pearson's *r* = 0.90, *P* = 2.0 × 10^−6^, Table S8). More importantly, correlations between ddPCR and FACS for untreated (Pearson's *r* = 0.50, *P* = 4.9 × 10^−3^) and PMAxx-treated fecal samples (Pearson's *r* = 0.39, *P* = 1.4 × 10^−1^, Table S8) were not stronger than correlations between qPCR and FACS (Table S3). Indeed, when quantifying serial 2-fold dilutions of 3 samples and mock mix (within the concentration range of ~10^2^-10^5^ copies/uL), we showed that qPCR and ddPCR results correlated strongly (Pearson's *r* = 0.988, *P* = 5.6 × 10^−46^ Table S9, Figure S5).

Altogether these results indicate that the deviant QMP-qPCR based profiles when compared to the other profiling methods cannot be explained by a lack of precision or sensitivity of qPCR.

In conclusion, our results show that quantitative microbial profiles are substantially affected by the method of microbial quantification.

Flow-cytometry counting excludes damaged cells and free extracellular DNA, while this part of the microbiome is being captured during sequencing. A significant part of bacterial death and lysis might occur during sample collection and handling in the laboratory, these bacteria should therefore not be dismissed during quantification. By using PMAxx treatment, we indeed demonstrate that on average around 40% of metagenomic DNA in fecal samples can be attributed to extracellular DNA and damaged bacterial cells. However, eliminating this part of metagenomic DNA prior to sequencing still resulted in highly similar quantitative microbiome profiles suggesting that bacterial cell death was evenly distributed across taxa. This indicates that extracellular DNA does not seem to introduce a new source of bias when combining 16S NGS with flow-cytometry cell counts. It should, however, be noted that a previous study did report markedly distinct fecal microbial profiles of extremely preterm infants upon PMA-treatment (Young et al., 2018). The rapid processing and storage of fecal samples in the present study might have contributed to the limited differences, underscoring the importance of careful sample handling.

The results of our analysis further demonstrate that quantification of bacterial load by qPCR results in highly divergent profiles, indicating that qPCR-based quantification might not be an adequate approach for quantitative microbiome profiling. Flow-cytometry quantification indicated that the difference in bacterial load varied <3 times between the vast majority of samples (14/16). Several studies have indicated that qPCR is only useful for determining dissimilarity between two samples if the true difference is at least 2–3-fold (Smith and Osborn, 2009; Hospodsky et al., 2010), suggesting that qPCR-based enumeration is too imprecise to be an adequate alternative for flow-cytometry in quantitative microbiome profiling. However, we showed that using the highly precise and sensitive ddPCR for microbial quantification did not result in improved correlation with flow cytometry-based cell counting. The strong correlation between ddPCR and qPCR moreover makes PCR bias an unlikely cause of the divergent profiles as different primer pairs were used for the two molecular quantification methods. Indeed*, in silico* analyses showed that the primer pairs used for qPCR quantification are highly specific for the domains of archaea and bacteria. Less that 0.1% of eukaryotic sequences are detected while over 95% of all bacterial sequences are being detected. Although only 65% of all archaeal sequences match our primer pair, this is mainly due to mismatches to many environmental archaea whereas the methanogenic archeal species commonly observed in the human intestinal tract are all covered by our primer pair.

When using Gut Microbiome Whole Cell Mix, we did show strong correlations between flow-cytometry and qPCR-based quantification. Moreover, ddPCR and qPCR quantification of 16S rRNA gene copies in fecal samples also correlated strongly despite the use of different primer pairs and amplification protocols. Together these results indicate that primer bias or other technical aspects related to qPCR-based quantification are an unlikely cause for the dramatic deviant QMP-qPCR profiles. It is much more likely that the bias is introduced during the process of extracting DNA from the complex fecal matrix. In contrast to cell counting, molecular quantification is a multi-step process on a small aliquot of the original fecal sample, which might result in increased intra-sample variation when performed on multiple aliquots. Indeed, the standard deviation between (some) replicates was substantially larger when using qPCR as compared to flow cytometry. This is in line with a recently published method to decompose spatiotemporal variance on microbial communities, which confirmed substantial heterogeneity between spatial sampling locations of fecal samples (Ji et al., 2019). Also, incomplete lysis and DNA fragmentation can bias results during DNA extraction, however the protocol used in the present study has been comprehensively optimized to maximize DNA quality and quantity and benchmarked to limit bias in community diversity and Gram-positive to Gram negative ratio (Costea et al., 2017). Moreover, the DNA extraction might also become saturated which even further hampers direct correlation between DNA yield and microbial load in the original sample. These limitations may also impact the use of alternative methods for quantitative profiling such as spiking in reference DNA as an internal standard to extrapolate the amount of starting nucleic material (Tkacz et al., 2018; Morton et al., 2019).

A previous study did report near perfect correlations between QMP-qPCR and absolute abundances as determined by various taxon-specific qPCRs (Jian et al., 2020). However, as both methods were applied on the same DNA sample this further suggests that the bias is not due to the qPCR-based approach itself but rather the lack of correlation between yield upon DNA extraction and the microbial load in the original fecal sample.

Flow-cytometry, being executed on the original sample, performed better in terms of intra-sample variations and showed stronger correlations with RMP and stool consistency. This suggests that flow-cytometry would be a more preferable method to quantify bacterial load in feces, however also flow-cytometry comes with several limitations. One such limitation is cell aggregation which can result in underestimation of cell counts (Gunasekera et al., 2000; Ou et al., 2017). Moreover, this method is more laborious, expensive and requires technical expertise (e.g., quality control and monitoring size-related resolution, setting-up reproducible scatter detection, measuring in accurate concentration range), which makes it less suitable as a standard method that can be applied by all labs on a high-throughput basis. This calls for more high-throughput and user-friendly cell-counting methods.

Alternatively, computational solutions are now becoming available to make stable inferences of changes in abundances in compositional data such as the application of “reference frames” (Morton et al., 2019). Such computational solutions should however always be accompanied by careful controlling for important confounding factors, in particular stool consistency.

In conclusion, quantitative microbiome profiling is an elegant approach to bypass the compositional nature of microbiome NGS data, however it is important to realize that technical sources of variability may introduce substantial additional bias depending on the quantification method being used.

## Data Availability Statement

The datasets presented in this study can be found in online repositories. The names of the repository/repositories and accession number(s) can be found at: https://www.ebi.ac.uk/ena, ERP108719.

## Ethics Statement

The studies involving human participants were reviewed and approved by Medical Ethics Committee of the Maastricht University Medical Center. The patients/participants provided their written informed consent to participate in this study.

## Author Contributions

This study was conceived by PS, PW, NB, LB, and JP. Experiments were designed by PS, JP, NB, CD, BB, KJ, PW, and ML. Sampling of healthy volunteers was set up and carried out by HB, CD, MO, and JP. Sample handling and processing was performed by CD and MO. Optimization of sequencing protocols and sequencing by NB and MH. PMAxx-treatment by ML and KJ. Flow cytometry by BB and PE. Statistical analyses by GG and JP. GG, NB, BB, and JP drafted the manuscript. All authors revised the article and approved the final version for submission.

## Conflict of Interest

The authors declare that the research was conducted in the absence of any commercial or financial relationships that could be construed as a potential conflict of interest.
