# Supplementary material for: How to Count Our Microbes? The Effect of Different Quantitative Microbiome Profiling Approaches
Source: Front Cell Infect Microbiol. 2020 Aug 7;10:403. doi: 10.3389/fcimb.2020.00403 (PMC7426659; doi:10.3389/fcimb.2020.00403)
Supplement: Supplementary file 2 [file Image_1.PDF]

Supplementary Material

Supplementary Figures

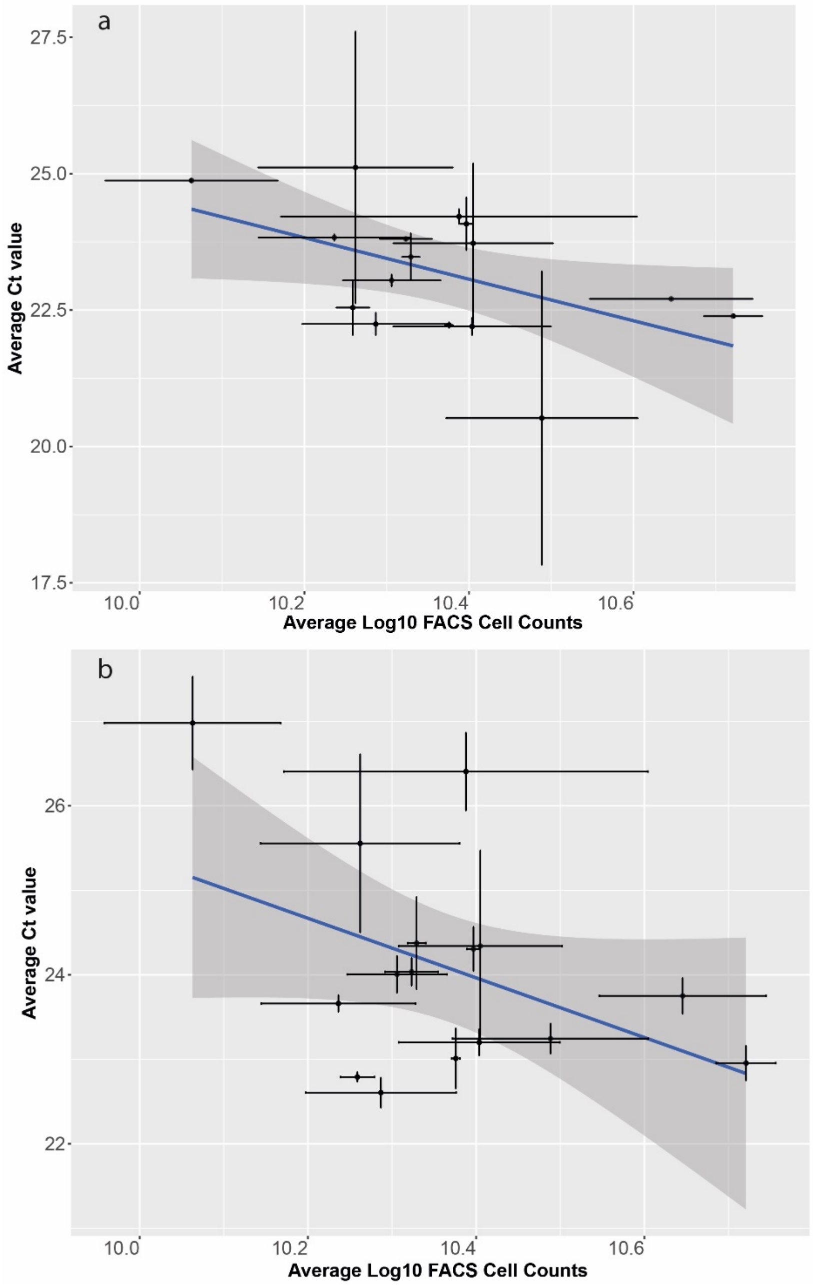

Figure S1. Quantification of microbial load by flow-cytometry and qPCR.

(a) Correlation between microbial loads as assessed by flow cytometry (log10 cell counts/gram faeces) and bacterial abundance as assessed by qPCR (n = 16 healthy subjects, Pearson's r = -0.50, P = 4.7 x 10^{-2}), **(b)** Correlation between microbial loads as assessed by flow cytometry (log10 cell counts/gram faeces) and bacterial abundance as assessed by qPCR (n = 16 healthy subjects) after treatment of faecal samples with PMAxx to remove non-viable cells and extracellular DNA (Pearson's r = -0.41, P = 1.1 x 10^{-1}). Data points represent median values of replicate samples, error bars represent the standard deviation.

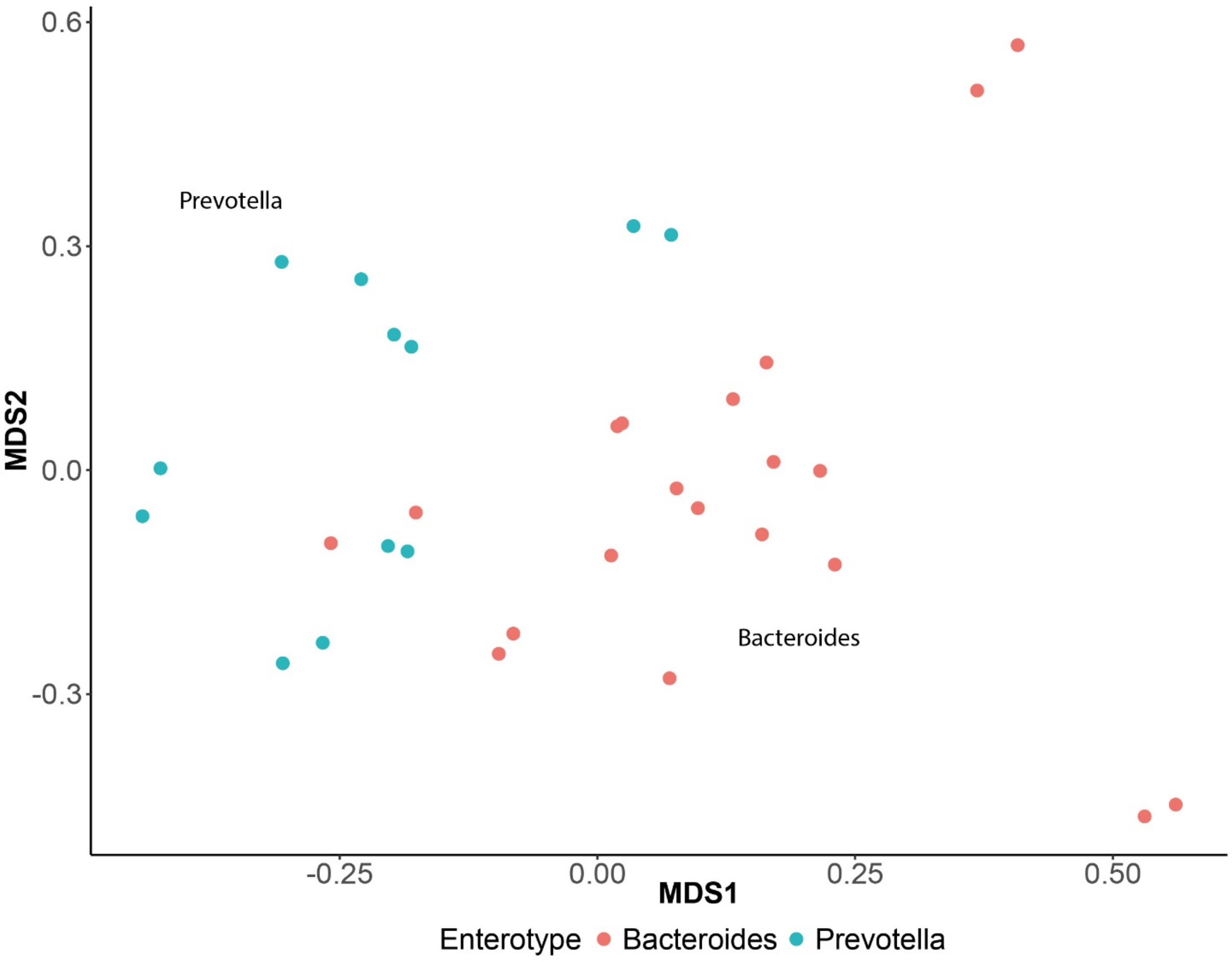

Figure S2. Genus-level faecal microbial community variation based upon Bray-Curtis dissimilarity and represented by non-metric Multidimensional Scaling. Both replicates from all 16 healthy individuals were included (n = 32), enterotyped based upon Dirichlet Multinomial Mixtures (DMM) and coloured accordingly.

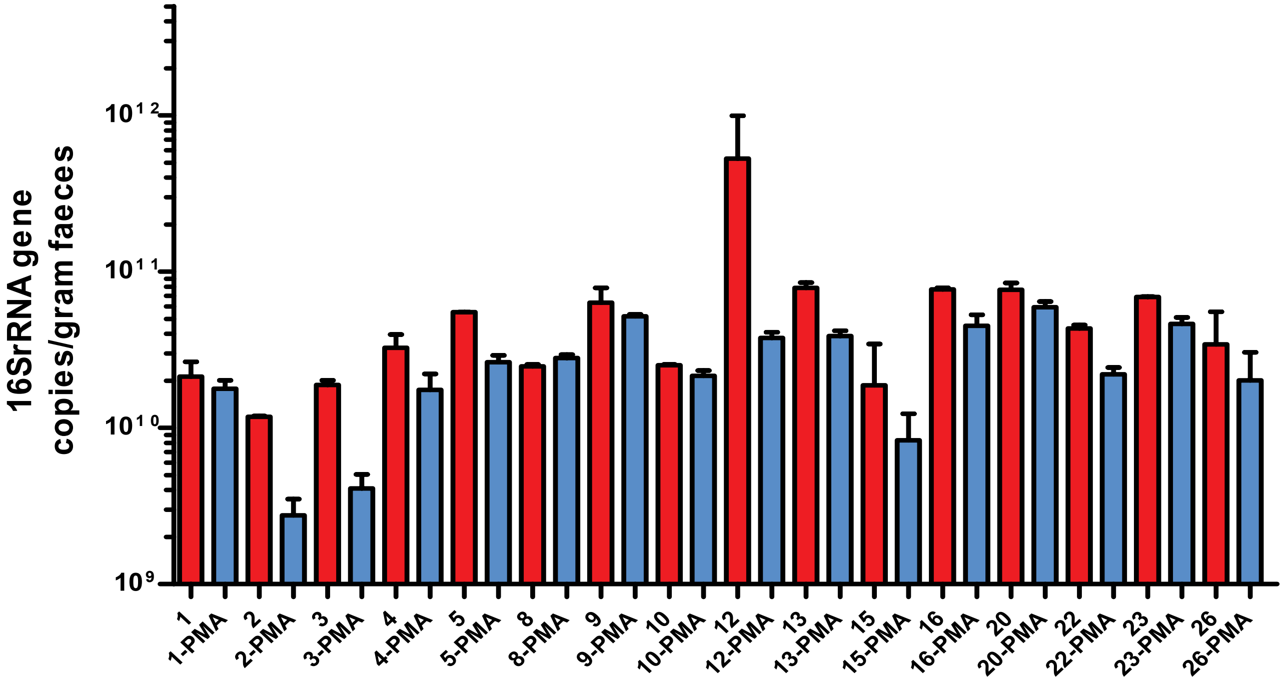

Figure S3. Average number of 16S rRNA gene copies per gram faeces for each sample before (red bars) and after (blue bars) treatment with PMAxx. Error bars indicate standard deviation based upon sample replicates.

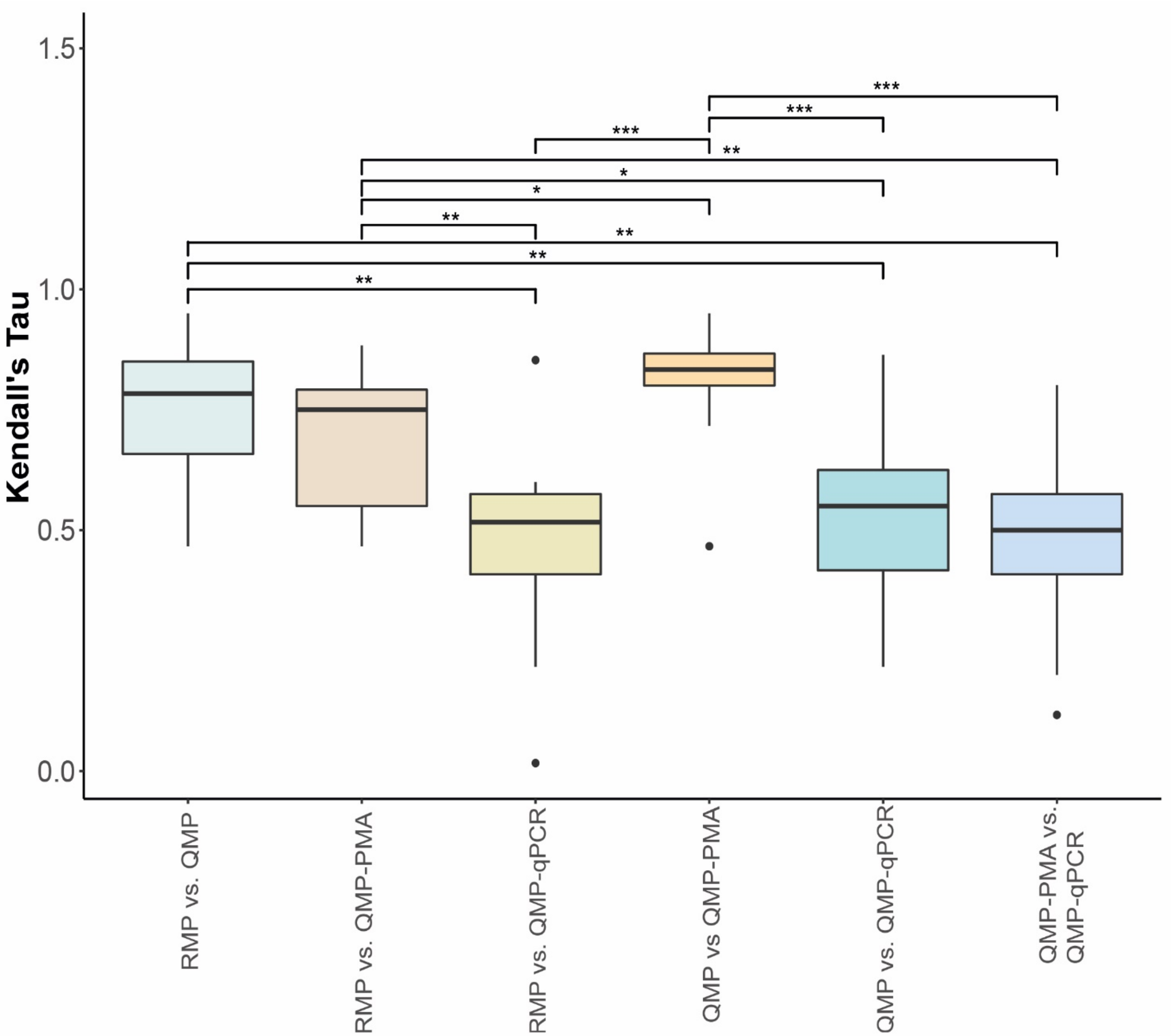

Figure S4. Boxplot based on the average Kendall's tau concordance between profiling methods among the ranked abundance of the 15 most abundant genera. The significance was checked pairwise using the Wilcoxon test and then adjusted for multiple comparisons using the FDR correction. The significance coding is indicated as *** for p<0.005, ** for p<0.01, and * for p<0. 05.

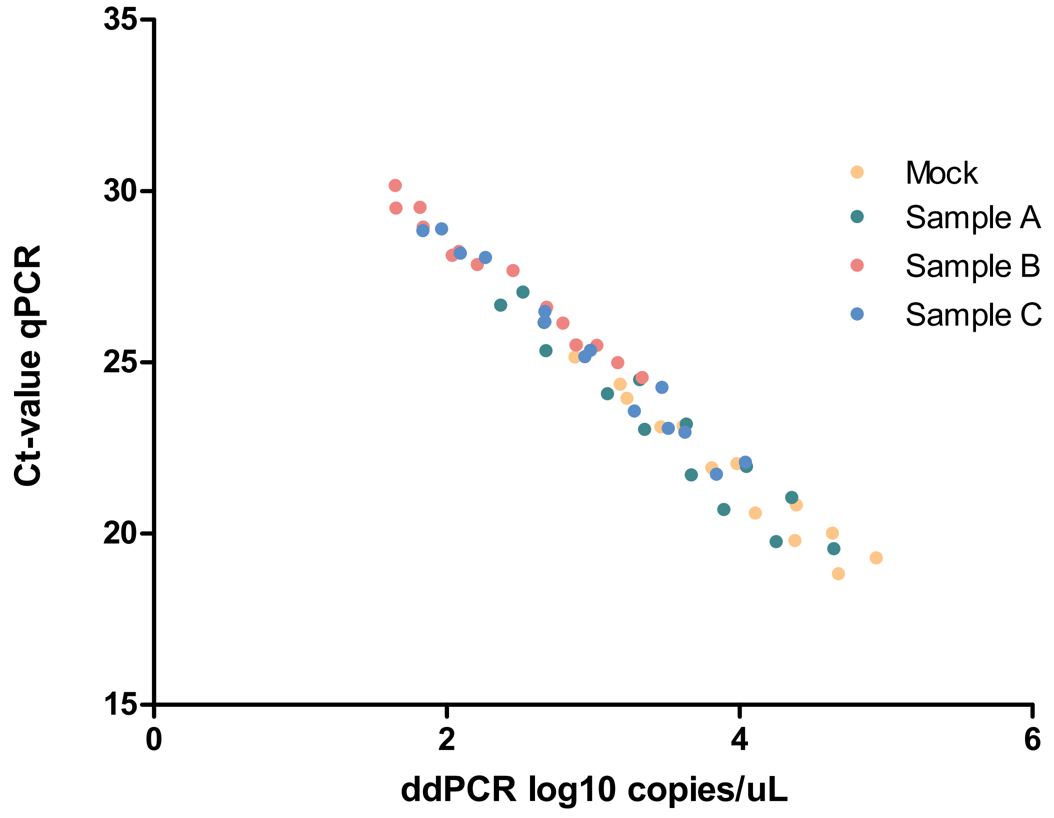

Figure S5. Scatter plot based on the quantification of serial 2-fold diluted mock and fecal samples by ddPCR as compared to qPCR. (Pearson's $r = -0.988$, $P = 5.6 \times 10^{-46}$)
